# Repressed SIRT1/PGC-1α pathway and mitochondrial disintegration in iPSC-derived RPE disease model of age-related macular degeneration

**DOI:** 10.1186/s12967-016-1101-8

**Published:** 2016-12-20

**Authors:** Nady Golestaneh, Yi Chu, Shuk Kei Cheng, Hong Cao, Eugenia Poliakov, Daniel M. Berinstein

**Affiliations:** 1 Department of Ophthalmology, Georgetown University Medical Center, 3900 Reservoir Road NW, Medical-Dental Building, Room NE203, Washington, DC 20057 USA; 2 Department of Neurology, Georgetown University Medical Center, Washington, DC USA; 3 Department of Biochemistry and Molecular & Cellular Biology, Georgetown University Medical Center, Washington, DC USA; 4 Retinal Cell and Molecular Biology (LRCMB), National Eye Institute, National Institutes of Health, Bethesda, MD USA; 5 Retina Group of Washington, Chevy Chase, MD 20815 USA

**Keywords:** AMD, RPE, Oxidative stress, Mitochondria, Cell viability, ROS, PGC-1α, SIRT1

## Abstract

**Background:**

Study of age related macular degeneration (AMD) has been hampered by lack of human models that represent the complexity of the disease. Here we have developed a human in vitro disease model of AMD to investigate the underlying AMD disease mechanisms.

**Methods:**

Generation of iPSCs from retinal pigment epithelium (RPE) of AMD donors, age-matched normal donors, skin fibroblasts of a dry AMD patient, and differentiation of iPSCs into RPE (AMD RPE-iPSC-RPE, normal RPE-iPSC-RPE and AMD Skin-iPSC-RPE, respectively). Immunostaining, cell viability assay and reactive oxygen species (ROS) production under oxidative stress conditions, electron microscopy (EM) imaging, ATP production and glycogen concentration assays, quantitative real time PCR, western blot, karyotyping.

**Results:**

The AMD RPE-iPSC-RPE and AMD Skin-iPSC-RPE present functional impairment and exhibit distinct disease phenotypes compared to RPE-iPSC-RPE generated from normal donors (Normal RPE-iPSC-RPE). The AMD RPE-iPSC-RPE and AMD Skin-iPSC-RPE show increased susceptibility to oxidative stress and produced higher levels of reactive oxygen species (ROS) under stress in accordance with recent reports. The susceptibility to oxidative stress-induced cell death in AMD RPE-iPSC-RPE and Skin-iPSC-RPE was consistent with inability of the AMD RPE-iPSC-RPE and Skin-iPSC-RPE to increase *SOD2* expression under oxidative stress. Phenotypic analysis revealed disintegrated mitochondria, accumulation of autophagosomes and lipid droplets in AMD RPE-iPSC-RPE and AMD Skin-iPSC-RPE. Mitochondrial activity was significantly lower in AMD RPE-iPSC-RPE and AMD Skin-iPSC-RPE compared to normal cells and glycogen concentration was significantly increased in the diseased cells. Furthermore, Peroxisome proliferator-activated receptor gamma coactivator 1-alpha (PGC-1α), a regulator of mitochondrial biogenesis and function was repressed, and lower expression levels of NAD-dependent deacetylase sirtuin1 (SIRT1) were found in AMD RPE-iPSC-RPE and AMD Skin-iPSC-RPE as compared to normal RPE-iPSC-RPE.

**Conclusions:**

Our studies suggest SIRT1/PGC-1α as underlying pathways contributing to AMD pathophysiology, and open new avenues for development of targeted drugs for treatment of this devastating neurodegenerative disease of the visual system.

**Electronic supplementary material:**

The online version of this article (doi:10.1186/s12967-016-1101-8) contains supplementary material, which is available to authorized users.

## Background

Age related macular degeneration (AMD) is a major cause of blindness in the developed countries, primarily affects the retinal pigment epithelium (RPE) resulting in subsequent degeneration of the photoreceptors [1–4]. AMD is a multifactorial disease with a complex interaction between environmental, metabolic and hereditary factors [5]. Clinically, AMD is presented in two forms, non-exudative and exudative. The non-exudative or dry form of AMD is diagnosed by polymorphic deposits, called drusen, that accumulate underneath the RPE and can result in overlying macular atrophy and pigmentation [6]. The exudative, or wet form of AMD is characterized by choroidal neovascularization leading to hemorrhage, retinal fluid, and eventual disciform scar formation [7]. However, an individual could present both forms at different stage of life, or initially develop dry form that would transform into wet form at later stage of the disease. To date, there is no effective treatment for dry AMD, yet millions of patients continue to lose their vision worldwide. Because of its complex etiology, understanding the molecular mechanisms of AMD has been challenging by lack of the appropriate in vitro model that could sufficiently recapitulate the characteristics of the disease. In addition, the available animal models fall short in accurately representing the characteristics of AMD due to absence of human genetic polymorphisms and long-term exposure to oxidative stress and environmental factors [8].

The generation of induced pluripotent stem cells (iPSCs) from somatic cells and their differentiation to various cell types offers new promise for autologous cell replacement therapies [9, 10]. These iPSCs also provide a prominent source for modeling diseases for which there is no adequate animal or in vitro model and may be used for in vitro drug screening [11]. Several groups have successfully differentiated RPE from iPSCs [12, 13] and we have demonstrated that iPSC-derived RPE are phenotypically and functionally similar to native RPE [14], thus offering promise for cell replacement therapy and disease modeling in AMD. A recent study has associated the abnormal *ARMS2/HTRA1* expression in iPSC-RPE from AMD patients with decreased SOD2 defense against oxidative stress making RPE more susceptible to oxidative damage [15]. Another study reprogrammed T cells from patients with dry type AMD into iPSCs-RPE and showed reduced antioxidant ability in AMD RPE as compared to normal RPE cells [16]. Recently, dysregulated autophagy in RPE was associated with increased susceptibility to oxidative stress and AMD [17, 18]. Another study related the decline in clearance system to induction of inflammasome signaling in human ARPE-19 cell line [19]. A more recent study reported mtDNA damage in RPE that may impact mitochondrial function [20]. However, to date, the phenotypic characterization of AMD patient-specific iPSC-RPE, as well as the underlying mechanisms responsible for the pathophysiology of AMD remains to be elucidated.

We cultured RPE from AMD and age-matched normal donors. Because primary RPE undergo senescence in culture by passaging, we generated iPSCs from the RPE of AMD and normal donor eyes with *CFH*, *HTRA1/ARMS2*, *LOC* abnormal alleles, or with *FACTOR B* protective alleles, followed by differentiation into RPE (AMD RPE-iPSC-RPE and Normal RPE-iPSC-RPE) (Table 1). We also generated iPSCs from skin fibroblasts of a dry AMD patient with *CFH*, *HTRA1/ARMS2*, *LOC*, and *FACTOR B* risk alleles, and differentiated them into RPE (Skin AMD iPSC-RPE) (Table 1). This approach allowed us to establish an inexhaustible in vitro disease model to study the molecular mechanisms of AMD.

**Table 1:** Genotyping and clinical information of AMD and control RPE, and patient’s skin fibroblasts from which the iPSC-RPE were generated

Donor ID #	Donor age-gender (M: male; F: female)	Clinical diagnosis	CFH (***C:risk***)	HTRA1 (***A:risk***)	LOC (***T:risk***)	Factor B (*T:protective*)	C2 (*C:protective*)	Smoking	Cause of death	Time of enucleation (h)	Method of reprogramming	Tissue of origin	Generated iPSC-RPE ID R: RPE-iPSC-RPE F:fibroblasts-iPSC-RPE
006	72-M	Control	***C***T	***A***G	G***T***	CC	GG	Quit in 1993	Chronic obstructive pulmonary disease	12	Sendai virus	RPE	6R
010	80-M	Control	***CC***	***A***G	G***T***	CC	GG	Quit in 1984	Acute myocardial infarction	9.4	Sendai virus	RPE	10R
025	50-M	Control	TT	GG	GG	CC	GG	No	Myocardial infarction	17	Sendai virus	RPE	25R
009	68-F	AMD	TT	GG	GG	*TT*	GG	2 ppd for 40 years	Stroke	3	Sendai virus	RPE	9R
032	75-M	AMD	***C***T	***AA***	***TT***	CC	GG	No	Pancreatic cancer	7	Sendai virus	RPE	32R

Patient ID	Patient age-gender (M: male; F: female)	Clinical diagnosis	CFH (***C:risk***)	FTRA1 (***A:risk***)	LOC (***T:risk***)	Factor B:(***A:risk***)	C2 (***A:risk***)	Smoking					
Patient genotype													
005BF	80-F	AMD	***CC***	***A***G	G***T***	***AA***	CC	Quit in 1982 10 cigarettes pd for 20 years	–	–	Sendai virus	Fibroblasts	005BF

The cause of death and time of enucleation are indicated. Cells were genotyped for known AMD-associated single nuclear polymorphisms (SNP) showing the haplotypes of each donor, carrying risk or protective alleles

A number of retinal pathologies including AMD are associated with mitochondrial dysfunction [21]. Dysfunctional mitochondria induce increased levels of ROS, mitochondrial DNA (mtDNA) damage, and defective metabolic activity [22]. A major role in mitochondrial biogenesis and oxidative metabolism is played by peroxisome proliferator-activated receptor-gamma coactivator (PGC)-1α (PGC-1α). Its repression contributes to disorders such as obesity, diabetes, neurodegeneration, and cardiomyopathy [23–27]. Recently DNA sequence variants in PPARGC1A gene coding for PGC-1α were reported to be associated with neovascular (NV) AMD and AMD-associated loci [28]. A more recent study reported a role for PGC-1α in induction of human RPE oxidative metabolism and antioxidant capacity [29]. PGC-1α is shown to play an important role in mitochondrial biogenesis and turnover [30, 31]; it also plays a role in autophagy/mitophagy in a manner that is specific to cellular metabolic state [32, 33]. In addition, PGC-1α is known to regulate the expression of electron transport chain (ETC) genes, lipid catabolism genes, and oxidative stress protective genes in vascular endothelial cells [34].

However, the role of PGC-1α in the pathophysiology of AMD remains to be elucidated. SIRT1 (silent information regulator T1) belongs to a family of class II histone/protein deacetylase proteins and is known as the only protein able to deacetylate and activate PGC-1α [35, 36]. SIRT1 has shown to play a major role in energy metabolism in various tissues that can directly interact and regulate the activity of transcription factors and co-regulators including PGC-1α [23].

We demonstrated that iPSC-derived RPE from RPE of AMD donors and from skin of an AMD patient exhibit specific disease phenotypes and impaired functions as compared to iPSC-RPE generated from RPE of normal donors. We sought to determine the underlying mechanisms responsible for the AMD disease phenotypes that could further direct us to development of new-targeted drugs for AMD.

## Methods

### Culture of RPE and fibroblasts

The eyes of organ donors clinically diagnosed with AMD and control organ donors were purchased from National Disease Research Interchange (NDRI, Philadelphia, PA). Eyes of donors with diabetes or other known ocular disease were excluded from the study. Eyes were enucleated on average 9 h after death and were delivered in less than 24 h. Serology tests were performed on each donor by NDRI to exclude samples with potential infectious diseases [37] (Table 1). RPE from macula of AMD donors and normal donors were isolated and cultured. Briefly, the posterior eyecup was dissected into four sections with a razor blade and was placed flat. The sensory retina was removed and the RPE-choroid layer was gently peeled. The RPE-choroid layer was digested by dispase, the RPE layer was peeled off with a forceps and filtered as reported previously [38]. RPE were purified with Magnetic-Activated Cell Sorting (MACS) by positive selection for epithelial cells using anti-BEST1 antibody (Abcam) and anti-E-cadherin (Miltenyi Biotech); and by negative selection using a fibroblast-specific antibody (Miltenyi Biotech) to remove fibroblasts. The RPE cells were grown in RPE Media [38] at 37 °C, with 5% O_2_ and 5% CO_2_.

Skin biopsy of the dry AMD patient was received in Viaspan preservation solution by courier immediately after the biopsy was taken. The skin biopsy was immediately treated upon reception for fibroblast isolation based on an established protocol [39] using DMEM containing 10% FBS at 37 °C, with 5% O_2_ and 5% CO_2_. Briefly, a small skin biopsy was incubated overnight in dispase solution. The next day the epidermis was peeled off with a forceps and the dermis was cut in small pieces and put in 24 well plates for fibroblasts to expand and grow as previously reported [39].

### Single nucleotide polymorphisms (SNPs) genotyping of RPE and fibroblasts

RPE from AMD and normal organ donors and fibroblasts from the AMD patient were tested for selected SNPs known to be associated with AMD by RT-PCR followed by sequencing analysis.

### Generation of iPSCs

The RPE from two AMD donors and three normal donors as well as fibroblasts from an AMD patient were used to generate iPSCs using non-integrating Sendai viruses according to the established protocol (CytoTune^®^-iPS Sendai Reprogramming Kits, ThermoFisher Scientific). The iPSCs were characterized by live staining of the colonies with Tra-1-60 antibody (LifeTechnologies), and fixed staining with Nanog antibody (Novus Biologicals). RT- PCR confirmed the expression of pluripotency genes.

### Differentiation of iPSCs to RPE

iPSCs were differentiated to functional RPE using the established protocol as previously described [14]. Briefly, the iPSCs were transferred to Aggrewells (Stem Cell Technologies; 1000–2000 cells/well) to form embryoid bodies (EBs) in suspension culture using Dulbecco’s modified Eagle’s medium (DMEM) with 10% serum replacement, 1× nonessential amino acids, 2 mM glutamine, and 0.1 mM b-mercaptoethanol) for 1 day, with 10 μM nicotinamide (NIC; SIGMA) added the next day and supplemented with 140 ng/ml recombinant human activin-A (R&D Systems, Minneapolis, MN, www.rndsystems.com) in the presence of NIC and SB431542 (SIGMA) for the 3rd and 4th week of differentiation. Pigmented foci were formed after the 4th week. Differentiating clusters were transferred to poly-d-lysine (2 lg/cm^2^, BD Biosciences) and 4 μg/ml laminin (SIGMA), for attachment on the 6th week and cultured in the presence of NIC for 3–5 weeks. Differentiating RPE cells were also cultured on 0.4 μm pore polyester membrane transwells (Corning) for induction of polarization and functional studies.

### Immunostaining

The iPSCs were cultured on 6-well plate laminine-coated dishes and stained either by live staining with Tra-1-60 antibody (Life Technologies) or fixed staining with Nanong antibody (Novus Biologicals) according to the manufacturer protocol.

The iPSC-RPE cells were cultured on transwells in 24-well plates and stained using primary and secondary antibodies as listed in the Additional file 1: Table S2. The iPSC-RPE were stained with anti ZO-1 antibody (Life Technologies), anti RPE65 (Generous gift from Dr. Redmond lab), anti Occludin antibody (Life Technologies), and anti Bestrophin antibody (Abcam) per manufacturer protocols. Anti-fading media were added to the cells before mounting onto glass slides (ThermoFisher, cat# P36930). Images were collected on EVOS FL microscope (Life Technologies) or confocal microscopy (Olympus Fluoview).

### Quantitative reverse transcription and polymerase chain reaction (qRT-PCR)

RNA was extracted from samples using the RNeasy Mini Kit (Qiagen, Germantown, MD). Samples were then treated with RNase-free DNase I and reversely transcribed using oligo-dT (SuperScript III cDNA synthesis kit from Qiagen, cat# 74104). Primers (Additional file 1: Table S1) used were specifically designed with PrimerQuest software (Integrated DNA Technologies).

### Immunobloting

IPSC-RPE cells were harvested from one well of 24 well plate. Cell pellets were homogenized in RIPA buffer by vortex every 10 min for 1 h. The homogenates were centrifuged at 15,000×*g* for 30 min at 4 °C, and the pellets were discarded. The protein concentrations of the supernatants were determined by the Bradford assay. The samples were then treated with NuPAGE LDS Sample Buffer (Cat. No. NP0007, Life Technologies), denatured by boiling for 5 min, and subjected to SDS-PAGE (NuPAGE 3–8% Tris-Acetate Gel, Cat. No. EA0378BOX, Invitrogen). The separated proteins were transferred to polyvinylidene difluoride membrane and the nonspecific binding sites were blocked by incubation with 5% nonfat dry milk in Tris buffered Saline, 0.01% Tween20 (TBST) for 1 h at room temperature. The membrane was then incubated with first antibody diluted in 5% bovine serum albumin (BSA)/TBST overnight at 4 °C. The β-actin antibody was used as a loading control. The immune-reactive bands were detected by Clarity Western ECL (BIO-RAD, cat# 1705060). Densitometry was performed using the ImageJ software.

### Phagocytosis of photoreceptor outer segments (POSs)

Bovine rod outer segments (POSs) were purified with discontinuous sucrose density gradient centrifugation as described previously [40]. The POS pellet was labeled with FITC using the established protocol [41].

Fluorescent POS (5 × 10^6^) were added in 40 μl of RPE media containing 2.5% sucrose to the apical surface of differentiated iPSC-RPE cells cultured on transwells; 40 μl/well for 4 h. The reaction was then placed on ice and rinsed four times with PBS containing 1 mM MgCl_2_ and 0.2 mM CaCl_2_ (PBS-CM). Samples were incubated in 0.2% Trypan blue in PBS-CM for 10 min to quench the reaction. Cells were fixed according to the protocol [41] and nuclei were labeled with DAPI. Images were collected on confocal microscopy (Olympus Fluoview).

### Electron microscopy

Cultured iPSC-RPE cells were first washed with Dulbecco PBS and then fixed 2.5% glutaraldehyde in PBS (pH 7.4) and 0.5% osmium tetroxide in PBS. The cells were then embedded in epoxy resin. 90 nm sections were collected on 200 μM copper mesh grids and left to dry for 24 h. The cells were then stained for uranyl acetate and lead citrate. JEOL JM-1010 electron microscope was used to view and image the cells.

#### Oxidative stress conditions

iPSC-RPE were cultured at 80–90% confluency in 96-well plates, at 37 °C with 5% O_2_ and 5% CO_2_. Oxidative stress was induced with different concentrations of H_2_O_2_ ranging from 0 to 10 mM for 24 or 48 h, followed by cell viability measurements. To test changes in gene expression levels under oxidative conditions, the iPSC-RPE were incubated with 0.4 mM of H_2_O_2_ for 2 h for 5 consecutive days.

### Cell viability assay

iPSC-RPE monolayer cells cultured in 96-well plates were incubated for 30 min with PrestoBlue Reagent containing a cell–permeant compound that is blue in color and nonfluorescent in solution. When added to media, the reagent is rapidly taken up by cells. The reducing environment within viable cells converts the reagent to an intensely red-fluorescent dye that can be detected by measuring fluorescence or absorbance (Life Technologies, cat# A13261). 6 wells were used for each sample. Fluorescence data were collected with 535 nm excitation wavelength and 612 nm emission wavelength (Ultra384 plate reader).

### Reactive oxygen species production assay

Measurements were carried out using OxiSelect™ Intracellular ROS Assay Kit (Cell Biolabs, cat# STA-342). Breifly, monolayers of iPSC-RPE cells were cultured in serum free RPE media for 20 h and then incubated for 1 h with 1 mM of cell permeable fluorogenic probe 2′-7′ Dichlorodihydrofluorescin diacetate (DCFH-DA). To induce oxidative stress, samples were incubated with 0.4 mM H_2_O_2_. The cell-permeable fluorogenic probe DCFH-DA diffuses into cells and is deacetylcated by cellular esterases into the non-fluorescent DCFH. In the presence of ROS, DCFH is rapidly oxidized to highly fluorescent DCF (Cell Biolabs, cat# STA-342). Tecan (Morrisville, NC) Ultra 384 plate reader measured the fluorescence intensity of each sample (which is proportional to ROS levels) against a standard for each given time point. Excell was used to calculate the ROS levels.

### Mitochondrial activity assay

ATP levels were used to assay the mitochondrial activities. Samples were incubated for 2 h with 10uM of bromopyruvate analogue (3-BrPA) (EMD Millipore, cat# 376817), an inhibitor of glycolytic hexokinase II enzyme. Measurements were collected with Mitochondrial ToxGlo Assay that is based on the differential measurement of biomarkers associated with changes in cellular ATP levels relative to vehicle-treated controls. The results are collected with bioluminescent readouts. Bioluminescent signal is proportional to ATP concentration (Promega, cat# G8000).

### Cytoplasmic glycogen concentration assay

Cytoplasmic glycogen levels were assayed using the Glycogen Assay Kit (Sigma, cat# MAK016) on iPSC-RPE monolayers grown in 96-well plates. Six wells were used for each sample. Glycogen concentration was determined by a coupled enzyme assay, which produces a colorimetric (570 nm)/fluorometric (535/587 nm) product, proportional to the glycogen present.

### G-band karyotyping of iPSC-RPE

G-band karyotyping of the iPSCs was performed according to the established protocol [42]. The iPSC-RPE were cultured in 25 cm^2^ dishes and prepared for karyotyping.

## Results

### Generation of functional iPSC-derived RPE

To investigate the molecular and cellular mechanisms of AMD, we generated iPSCs from RPE of AMD and age-matched normal donor eyes (RPE-iPSCs) and from skin fibroblasts of a dry AMD patient (Skin-iPSCs) (Table 1). While primary RPE could be used to study the disease phenotypes in AMD, they can quickly become depleted due to passaging and undergo senescence, whereas, iPSCs can serve as an inexhaustible source that could continuously be differentiated to the RPE for maintenance of the disease model.

We purified and cultured the RPE isolated from the macular region of the human eyes according to the established protocol [38] and performed the genetic study of the single nucleotide polymorphisms (SNPs) for the known AMD susceptibility loci. Table 1 summarizes the age, gender, and genetic characteristics of the cultured RPE from donors and the skin fibroblasts of an AMD patient. As shown in Table 1, the control RPE #010 exhibits SNPs for known AMD susceptibility loci, however the donor did not present AMD at 80 years old. Moreover, the AMD RPE #009 with a history of long-term heavy smoking while exhibiting the protective alleles, presented AMD pathology. This further reinforces that in addition to genetic background, environmental and epigenetic factors also play important roles in the etiology of AMD and that the susceptibility alleles are not the sole contributors to the disease.

For generation of iPSCs we used non-integrating Sendai viruses. The generated iPSC colonies were manually picked and expanded and were characterized by staining with Tra1-60 and Nanog (Additional file 1: Figure S1A) as well as by Real-Time PCR for pluripotency markers (Additional file 1: Figure S1B). To verify that the iPSCs do not present chromosomal abnormalities, karyotyping was performed on all generated iPSC lines. G band karyotyping analyses are presented in Additional file 1: Figure S2. As shown in Additional file 1: Figure S2, all tested cells for the AMD RPE-iPSC-RPE and the AMD Skin-iPSC-RPE with female background present normal karyotyping with XX chromosomes (9R, 32R and 005BF). The healthy RPE-iPSC-RPE with male background were normal for all somatic chromosomes, however, in approximately 50% of the tested cells the Y chromosome was missing. It is known that loss of the Y chromosome in males is a phenomenon associated with aging [43], and it is quite probable that the donors presented aneuploidy prior to sample collections. This is further supported by reports showing that the predominant genetic changes found in hiPSC lines involve changes in chromosomes 1, 12, 17 and 20, reminiscent of the changes observed in cancer cells [44].

**Fig. 1:**
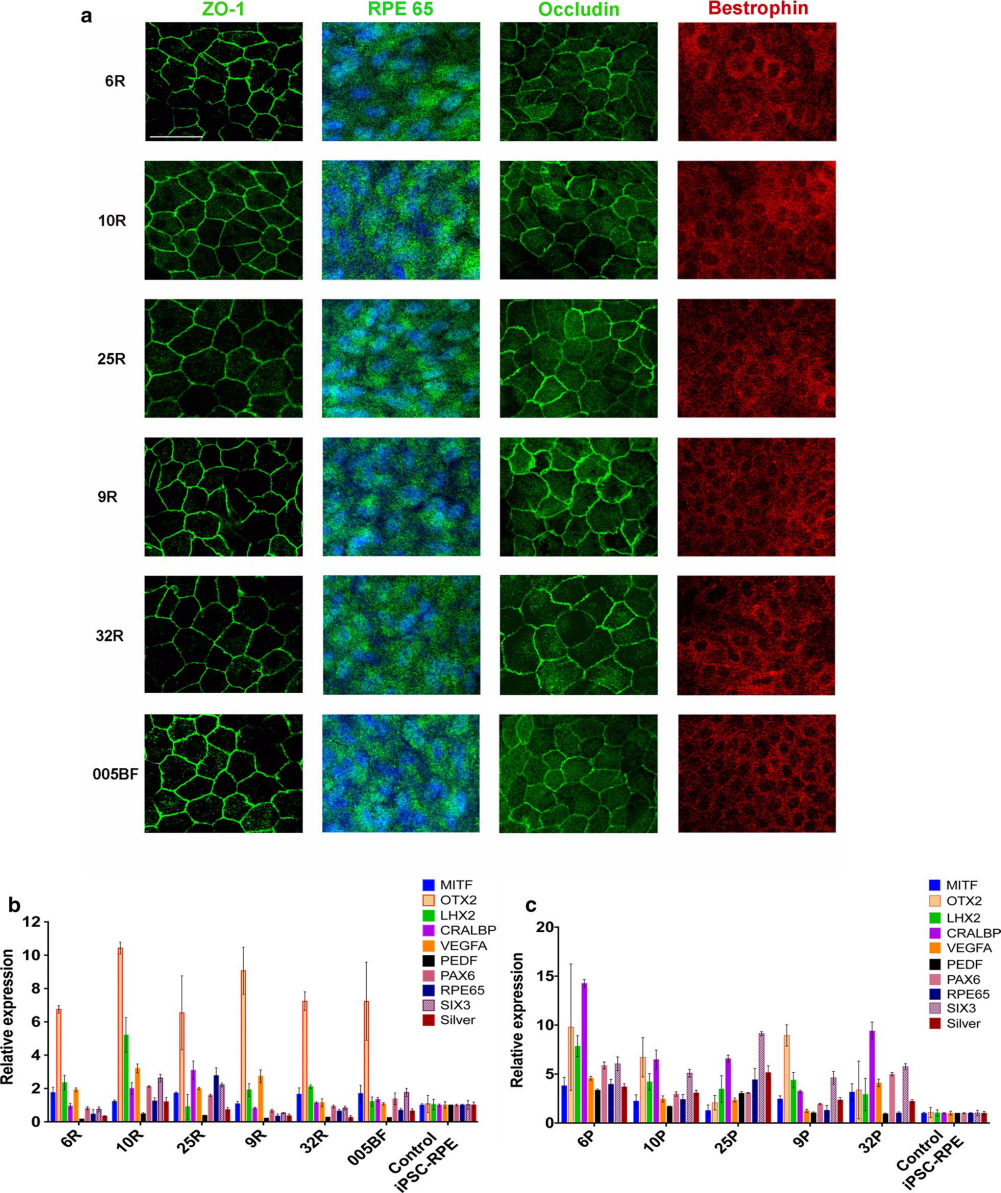
Differentiation and characterization of iPSC-RPE. **a** Immunostaining of the iPSC-RPE cultured on transwells for 4 weeks with ZO-1 antibody, RPE65, Occludin, and Bestrophin. *Scale bar* represent 100 μM. **b** Graph illustrating RPE specific gene expressions in differentiated iPSC-RPE cell lines. **c** RPE specific gene expression in native RPE from which the iPSC-RPE are generated. Relative expression of each gene to GAPDH is compared to its relative expression level in control iPSC-RPE

The iPSCs were differentiated into functional RPE as previously described [14], and the generated iPSC-RPE cell lines were characterized by immunostaining with ZO-1, RPE65, Occludin and Bestrophin antibodies (Fig. 1a). Our data showed that all iPSC-RPE cell lines were positive for the above staining and were characterized as RPE.

We also performed real-time PCR for RPE signature genes with iPSC-RPE and their parental donors the primary RPE. All iPSC-RPE expressed the RPE specific genes comparable to the primary RPE from which they were derived (Fig. 1b,c).

**Fig. 2:**
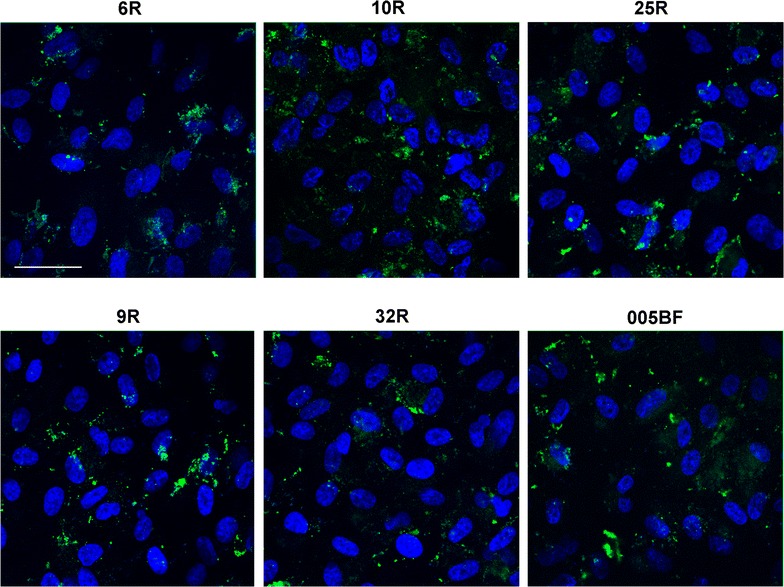
Phagocytosis assay in iPSC-RPE. Phagocytosis assay on iPSC-RPE cultured on transwells for 4 weeks representing internalized FITC-conjugated POS. The reaction was quenched with Trypan blue to mask uninternalized POS. Nuclei are represented in *blue* by DAPi staining. *Scale bar* represent 100 μM

To confirm the functionality of the iPSC-RPE we performed phagocytosis assay by incubating the iPSC-RPE with bovine photoreceptor outer segments (POS) that were conjugated with FITC. We quenched the uninternalized POS by treating the samples with Trypan blue to only visualize the internalized POS. Figure 2 shows the POS phagocytosis in iPSC-RPE cultured on transwells for 4 weeks, and reveal that all cell lines are functional, capable of phagocytosing the POS. A representative image for normal and AMD samples with and without Trypan blue treatment is shown in Additional file 1: Figure S3.

### Identification of distinct functional impairments in AMD RPE-iPSC-RPE and AMD Skin-iPSC-RPE

**Fig. 3:**
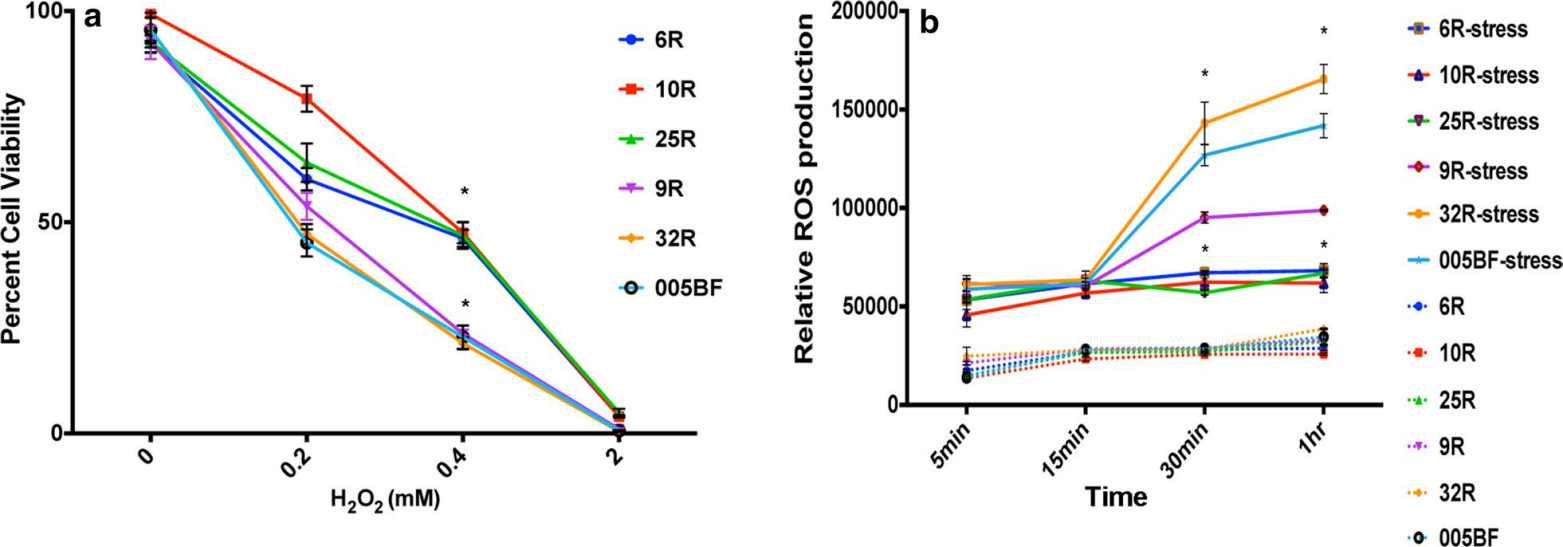
AMD iPSC-RPE exhibit increased susceptibility to oxidative stress and produce higher ROS. **a** Cell viability assays of AMD and control iPSC-RPE treated with increasing concentrations of H_2_O_2_ for 48 h. Higher susceptibility to oxidative stress-induced cell death under oxidative stress conditions (0.1, 0.2 and 0.4 mM H_2_O_2_) is observed in AMD RPE-iPSC-RPE (9R, 32R) and AMD Skin-iPSC-RPE (005BF) as compared to normal RPE-iPSC-RPE (6R, 10R, 25R). **b** ROS production under stress conditions is significantly higher in AMD RPE-iPSC-RPE (9R, 32R) and AMD Skin-iPSC-RPE (005BF) as compared to normal RPE-iPSC-RPE (6R, 10R, 25R). *Asterisk* (*) in **a** and **b** indicates statistically significant difference between control and AMD iPSC- RPE, determined by student *t* test, p ≤ 0.05

**Fig. 4:**
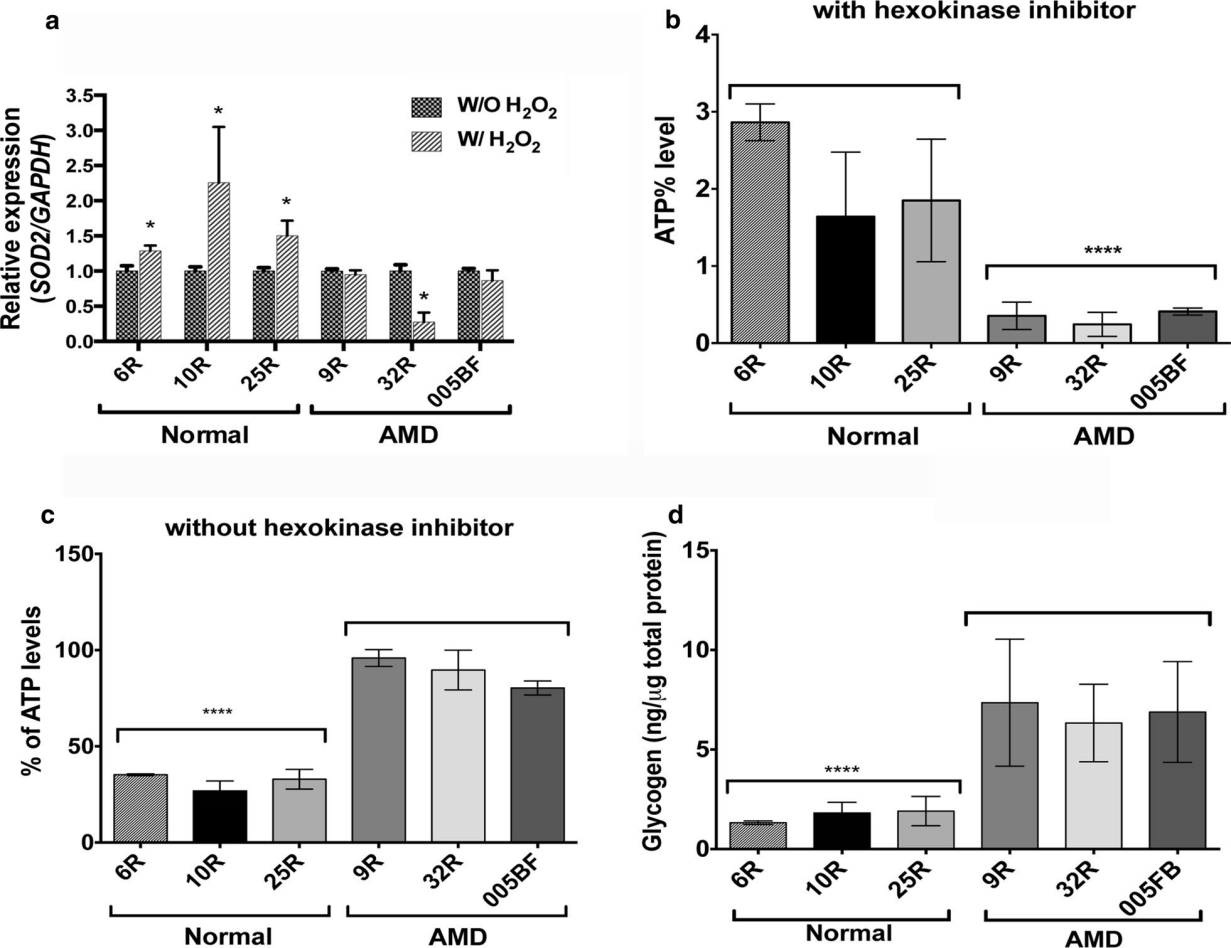
AMD iPSC-RPE express lower SOD2 defense, lower mitochondrial activity and present higher cytoplasmic glycogen concentration. **a** AMD RPE-iPSC-RPE and AMD Skin-iPSC-RPE are not capable of increasing *SOD2* expression under stress conditions as compared to normal RPE-iPSC-RPE. AMD and control iPSC-RPE were treated with 0.4 mM H_2_O_2_ for 2 h for 5 consecutive days after which RNA were extracted and analyzed via quantitative RT-PCR. As opposed to normal RPE-iPSC-RPE (6R, 10R, 25R), the AMD RPE-iPSC-RPE (9R, 32R) and AMD Skin-iPSC-RPE (005BF) were not responsive to stress conditions and did not increase the *SOD2* gene expression under stress conditions. GAPDH was used to normalize the samples and the relative expression of each sample is compared. *Asterisks* (*) in **a** indicate statistical significance, determined by student *t* test (p ≤ 0.05). **b**, **c** AMD iPSC-RPE have significantly lower mitochondrial activity as compared to control iPSC-RPE, as indicated by ATP levels measured by a luminescence assay in the presence (**b**) and absence (**c**) of hexokinase inhibitor. *Asterisks* (***) in **b** and **c** show significance analyzed by Anova followed by Tukey, p ≤ 0.0001. **d** Measurement of cytoplasmic glycogen accumulation by colorimetric assay showing higher concentration in AMD iPSC-RPE as compared to control iPSC-RPE. *Asterisks* (****) in **d** show significance analyzed by Anova followed by Tukey, p ≤ 0.0001

We have differentiated the AMD and normal RPE-iPSCs, and AMD Skin-iPSC to functional RPE, we then investigated their phenotypic and functional characteristics . Interestingly, both AMD RPE-iPSC-RPE and AMD Skin-iPSC-RPE demonstrated similar disease-relevant cellular phenotypes, whereas, the normal RPE-iPSC-RPE were devoid of disease phenotypes, and presented normal cellular and molecular characteristics (Figs. 3, 4).

We performed several cellular and molecular assays. First, we verified the cell viability of the AMD RPE-iPSC-RPE and AMD Skin-iPSC-RPE as compared to normal iPSC-RPE under oxidative stress conditions that we have established in our lab by incubating the RPE under increasing concentration of H_2_O_2_ for 48 h. Cell viability assay under oxidative stress conditions revealed that the AMD RPE-iPSC-RPE and AMD Skin-iPSC-RPE show increased susceptibility to oxidative stress and present higher levels of cell death at 0.1, 0.2 and 0.4 mM of H_2_O_2_ when compared to normal RPE-iPSC-RPE under the same conditions (Fig. 3a).

To test whether the AMD RPE-iPSC-RPE and AMD Skin-iPSC-RPE exhibit increased reactive oxygen species (ROS) production, we quantified the ROS production in AMD RPE-iPSC-RPE and AMD Skin-iPSC-RPE compared to normal iPSC-RPE in the presence of 0.4 mM of H_2_O_2_ for 0, 5, 15, 30 min and 1 h incubation. Our results shown in Fig. 3b revealed that AMD RPE-iPSC-RPE and AMD Skin-iPSC-RPE produce significantly higher levels of ROS under oxidative stress conditions as compared to normal RPE-iPSC-RPE.

These observations are in accordance with the results showed by Chang et al. [16]. We also demonstrate that both AMD RPE-iPSC-RPE and AMD Skin-iPSC-RPE exhibit the disease relevant cellular phenotype and could be used for in vitro disease modeling in AMD.

Super oxide dismutases (SODs) provide defense against ROS and plays an important role in controlling oxidative stress [45]. To investigate the involvement of *ARMS2/HTRA1* in affecting the SOD2 defense in RPE as reported by Yang et al., we measured the expression levels of superoxide dismutase 2 (SOD2) in AMD RPE-iPSC-RPE and AMD Skin-iPSC-RPE compared to normal RPE-iPSC-RPE by Real-Time PCR under normal and our established chronic stress conditions by incubation of the cells for 2 h with H_2_O_2_ at 0.4 mM for 5 consecutive days.

Our data showed that only the normal RPE-iPSC-RPE cells harboring either abnormal *ARMS2/HTRA1* allele (6R, 10R) or normal *ARMS2/HTRA1* allele (25R) were able to increase the *SOD2* expression during stress conditions, whereas the *SOD2* expression levels were not increased in AMD RPE-iPSC-RPE and AMD Skin-iPSC-RPE with abnormal *ARMS2/HTRA1* allele (32R, 005BF), or in AMD RPE-iPSC-RPE with normal *ARMS2/HTRA1* and protective *Factor B* alleles (9R, a heavy smoker donor) under the same conditions (Fig. 4a). This inability to increase the *SOD2* levels under stress conditions correlates with the increased susceptibility to oxidative stress-induced cell death observed in the AMD RPE-iPSC-RPE and AMD Skin-iPSC-RPE (Fig. 3a). These observations further suggest that besides the AMD risk alleles, other unknown factors such as genetic, environmental, or epigenetic factors may play a role in regulating SOD2 defense levels and in AMD pathophysiology.

It has been reported that damaged mitochondria leads to increased ROS production by the cells [46]. Since SOD2 is a mitochondrial protein that plays an important role in antioxidant defense, we sought to investigate the mitochondrial activity in AMD RPE-iPSC-RPE and AMD Skin-iPSC-RPE. The mitochondrial activity was evaluated by measurement of ATP production in the presence and absence of hexokinase inhibitor that inhibits the ATP produced by glycolysis. Our data showed that ATP production in the presence of hexokinase inhibitor that solely represents mitochondrial ATP production, was significantly reduced in AMD RPE-iPSC-RPE and AMD Skin-iPSC-RPE compared to normal RPE-iPSC-RPE (Fig. 4b); whereas, the total ATP production in the absence of hexokinase inhibitor was higher in AMD RPE-iPSC-RPE and AMD Skin-iPSC-RPE compared to normal RPE-iPSC-RPE, suggesting that the majority of ATP in the AMD RPE-iPSC-RPE and AMD Skin-iPSC-RPE is produced by glycolysis (Fig. 4c).

Enhanced glycogenesis is associated with cellular senescence [47] and glycogen accumulation occurs in diverse cellular senescence models [47]. To test whether glycogen accumulation is a cellular phenotype in AMD RPE-iPSC-RPE and AMD Skin-iPSC-RPE, we determined the cellular glycogen concentration. Interestingly, glycogen concentration was significantly higher in AMD RPE-iPSC-RPE and AMD Skin-iPSC-RPE when compared to normal RPE-iPSC-RPE (Fig. 4d).

The susceptibility to oxidative stress, higher levels of ROS, increased glycogen concentration and inability of AMD RPE-iPSC-RPE and AMD Skin-iPSC-RPE to increase antioxidant defense can be explained by dysfunctional mitochondria observed in our AMD iPSC-RPE cells.

### Identification of disease-relevant cellular phenotypes in AMD RPE-iPSC-RPE and AMD Skin-iPSC-RPE

**Fig. 5:**
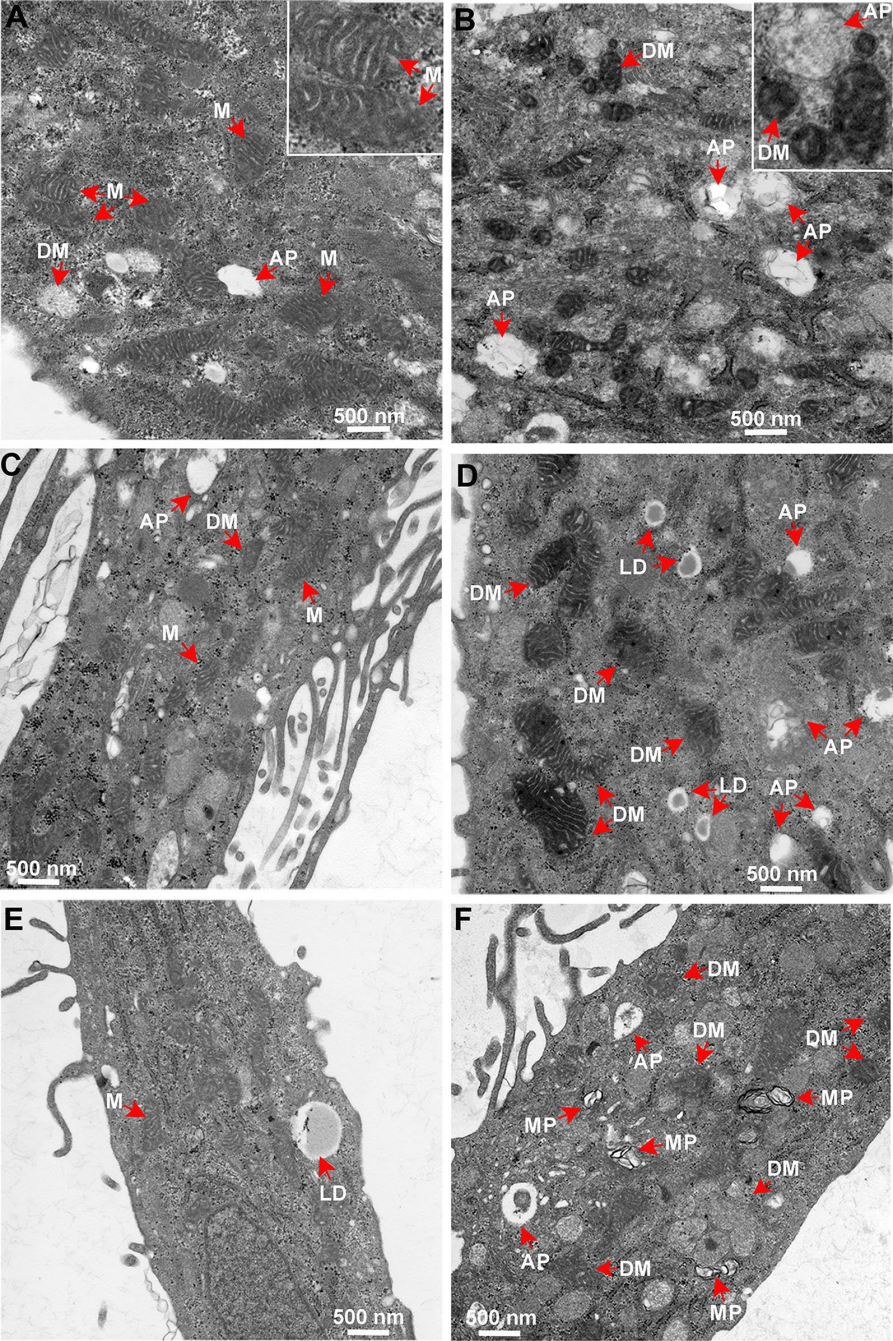
AMD iPSC-RPE exhibit relevant cellular phenotypes. **a**–**f** Representative electron microscopy images of normal (**a** 25R; **c**10R; **e** 25R), AMD-RPE-iPSC-RPE (**b** 32R; **f** 9R) and AMD Skin iPSC-RPE (**d** 005BF) showing diseased phenotypes. *Red arrows* indicate the observed morphological differences. Higher magnification insets show disintegrated mitochondria (in **b** 32R), in comparison with the normal mitochondria (in **a** 25R). *M* mitochondria, *DM* disintegrated mitochondria, *LD* lipid droplets, *AP* autophagosomes. All *scale bars* represent 500 nm

Our data from functional assays lead us to phenotypical analysis of the AMD RPE-iPSC-RPE and AMD Skin-iPSC-RPE compared to normal iPSC-RPE. Figure 5a–f show the electron microscopy imaging (EM) of the diseased and normal iPSC-RPE. As shown in Fig. 5, the AMD RPE-iPSC-RPE (b, f) and AMD Skin-iPSC-RPE (d) appear to have disintegrated mitochondria, increased number of autophagosomes and higher levels of cytoplasmic lipid accumulation (f) as compared to normal RPE-iPSC-RPE (a, c, e).

Dry AMD is characterized by accumulation of lipid deposits called drusen between the RPE and Bruch’s membrane [6]. In addition, mitochondrial dysfunction is shown to induce formation of lipid droplets as a response to stress [48, 49]. Therefore, accumulation of lipid droplets in AMD-iPSC-RPE could reflect a disease-relevant cellular phenotype. These phenotypic observations correlate with the susceptibility of the AMD iPSC-RPE to oxidative stress, higher production of the ROS and inability of the AMD iPSC-RPE to increase *SOD2* levels under the stress conditions and reduced mitochondrial activity in AMD iPSC-RPE.

### Repressed SIRT1/PGC-1α pathway in AMD RPE-iPSC-RPE and AMD Skin-iPSC-RPE

**Fig. 6:**
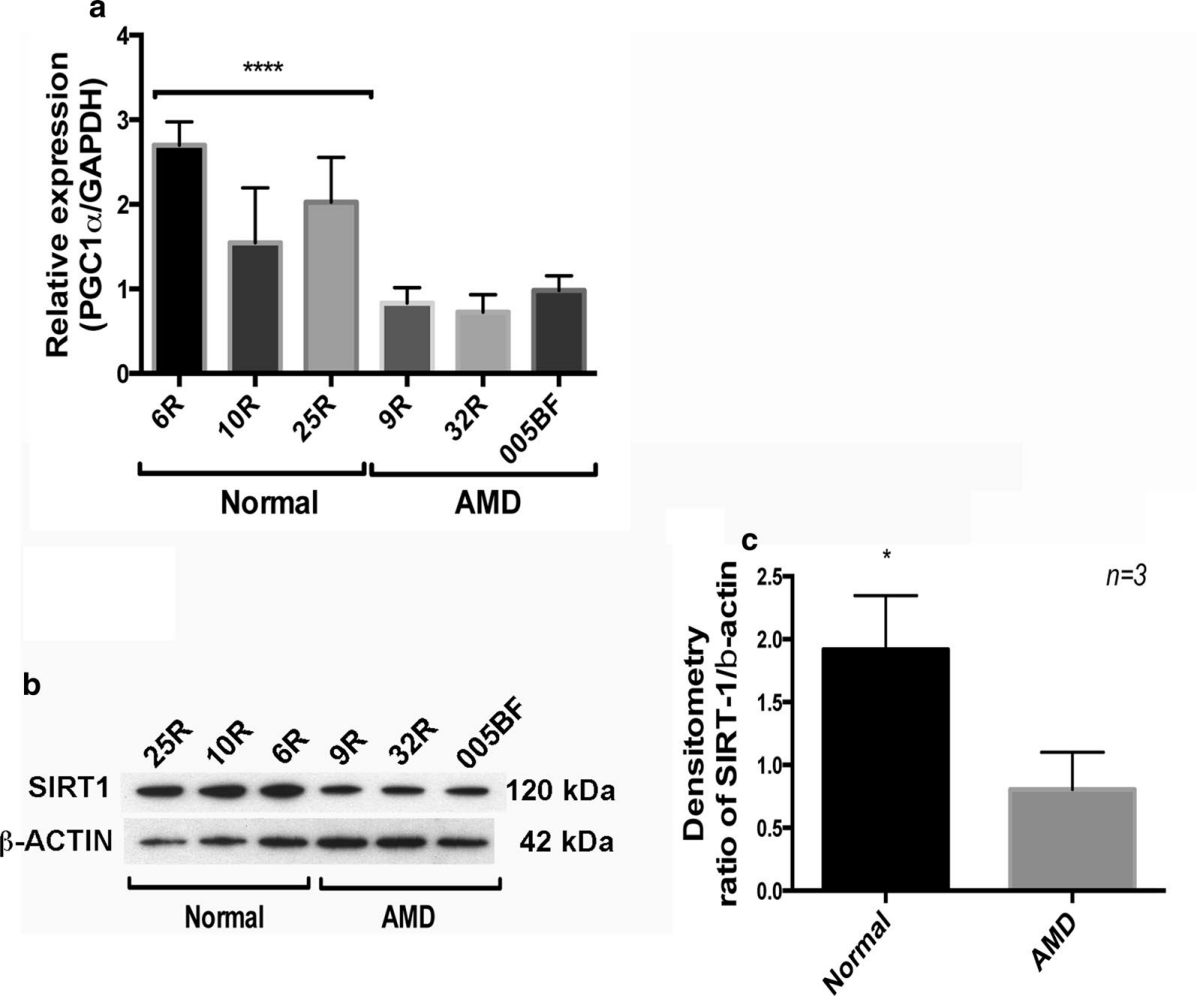
Reduced SIRT1 protein levels and *PGC*-*1* expression in AMD-iPSC-RPE. **a** Quantitative real time PCR showing decreased *PGC*-*1* expression in AMD-iPSC-RPE as compared to normal iPSC-RPE. *Graph* represents mean ± SE of three independent experiments. *Asterisk* (****) show statistically significance analyzed by Anova followed by Tukey, p ≤ 0.0001. **b** Representative western blot image of three independent experiments showing SIRT1 protein levels in iPSC-RPE. B-actin was used as normalization control. **c** Densitometry of average of three independent western blots showing ≅twofold decrease in SIRT1 protein levels in AMD RPE-iPSC-RPE and AMD Skin-iPSC-RPE as compared to normal RPE-iPSC-RPE. *Asterisks* (*) in **c** show significance analyzed by Anova followed by Tukey, p ≤ 0.05

Based on our observations on mitochondrial disintegration (Fig. 5b–f) and dysfunction (Fig. 4b, c), and the reduced ability to induce SOD production under oxidative stress in AMD RPE-iPSC-RPE and AMD Skin-iPSC-RPE (Fig. 4a), we sought to investigate the implication of the pathways regulators of mitochondrial biogenesis and functions. We first tested the expression of PGC-1α by real time PCR. While PGC-1α gene expression could vary from sample to sample, we observed a general trend of reduced PGC-1α exression levels in our AMD RPE-iPSC-RPE and AMD-Skin-iPSC-RPE as compared to normal RPE-iPSC-RPE (Fig. 6a).

Since SIRT1 acts upstream of PGC-1α and regultaes PGC-1α activity [50], we next analyzed the SIRT1 protein expression by western blot analysis in all generated cells lines. Our data showed a decrease in SIRT1 protein levels in AMD RPE-iPSC-RPE and AMD-Skin-iPSC-RPE as compared to normal RPE-iPSC-RPE (Fig. 6b). Densitmetry analysis of three independent experiements showed ≅twofold decrease in SIRT1 protein in our AMD RPE-iPSC-RPE and AMD Skin-iPSC-RPE as compared to normal RPE-iPSC-RPE (Fig. 6c).

These observations suggest dysfunctional SIRT1/PGC-1α pathway as the underlying mechanisms contributing to AMD pathophysiology and correlate with the disease cellular phenotypes that we observed in our AMD iPSC-RPE.

## Discussion

AMD is a complex multifactorial disease that is caused by contribution of genetic, metabolic and environmental factors. Millions of people worldwide are suffering from AMD and the prevalence of the disease is expected to double by 2050 [51]. The molecular mechanisms of AMD remain poorly understood due to the lack of a human in vitro model that exhibits the characteristic of the disease. While several animal models are being studied, they do not fully recapitulate the complex multifactorial aspects of AMD. Moreover, the single nucleotide polymorphisms and life-long exposure to oxidative stress in human make the study of AMD even more challenging.

The iPSC technology has become a revolutionized method to model diseases for which there is no adequate human in vitro model. While iPSC-derived disease models have been successfully generated and characterized in monogenic diseases, generation of in vitro disease models with significant cellular phenotypes in multifactorial diseases has been less successful [11, 52]. Several studies have shown that iPSC retain the epigenetic memory of their tissue of origin [53–57], while environmental and epigenetic causes of diseases are erased by reprogramming [52]. Another study by Hu et al. has reported that reprogrammed human RPE cells show tendency for spontaneous redifferentiation into RPE, suggesting that the epigenetic memory is retained in iPSCs [54]. These observations suggest that RPE-iPSC-RPE might be superior to Skin-iPSC-RPE for AMD disease modeling. To address this point, here we have generated iPSCs from RPE of macular region of healthy donors and donors clinically diagnosed with AMD, and from skin biopsy of a dry AMD patient, followed by differentiation into RPE. We then characterized them and performed functional studies. Our data revealed that both cell types exhibit similar disease relevant phenotypes and are prominent sources for AMD disease modeling. We demonstrate that patient-specific iPSC-RPE exhibit distinct disease phenotypes compared to normal iPSC-RPE and could be an excellent source for disease modeling and for development of new treatments for AMD. However, these findings should be taken into consideration prior to attempts for autologous cell transplantation in AMD.

We observed relevant disease phenotypes, such as susceptibility to oxidative stress and increased levels of ROS formation in accordance with the recently reported data [16]. We also observed lower SOD2 defense in AMD iPSC-RPE with abnormal *ARMS2/HTRA1* expression concomitant with the recent report by Yang et al. [15]. However, we also found that the iPSC-RPE generated form RPE of normal donor harboring abnormal *ARMS2/HTRA1* expression (Table 1), had the ability to increase *SOD2* expression under oxidative stress condition, whereas the AMD iPSC-RPE generated from RPE of AMD donor with normal *ARMS2/HTRA1* and with protective *FACTOR B* allele but with a history of heavy-smoking presented reduced ability to increase SOD2 levels under the same stress conditions. These observations further support the multifactorial origin of AMD and suggest that the susceptibility alleles may not be the sole contributor to lowered SOD2 defense and support our observations that repressed PGC-1α/SIRT1 pathway could contribute to AMD pathophysiology.

Mitochondria is known to be the major source of ROS production and an excess of ROS can induce mitochondrial damage and lead to diseases [58, 59]. Mitochondrial dysfunction is reported to induce formation of lipid droplets as a generalized response to stress [48], and to cause a reduction in mitochondrial oxidative and phosphorylation activity [60]. We therefore measured mitochondrial activity in AMD RPE-iPSC-RPE and AMD Skin-iPSC-RPE and compared it to normal iPSC-RPE-RPE. Our data revealed decreased ATP production by mitochondria and increased ATP production by glycolysis in AMD RPE-iPSC-RPE and AMD Skin-iPSC-RPE compared to normal RPE-iPSC-RPE. This finding suggests glycolysis as the major source of ATP production in AMD RPE-iPSC-RPE and AMD Skin-iPSC-RPE. While ATP is the major source of cellular energy, elevated levels of ATP can inhibit AMP-activated protein kinase (AMPK), a key energy sensor that regulates cellular metabolism and autophagy [61, 62]. This could explain the accumulation of autophagosomes that we observed in AMD RPE-iPSC-RPE and AMD Skin-iPSC-RPE, which indicates inhibition of autophagic dynamics, and accumulation of unwanted and undigested materials in the cells.

Phenotypic analysis by EM revealed disintegrated mitochondria, accumulation of autophagosomes and lipid droplets in AMD RPE-iPSC-RPE and AMD Skin-iPSC-RPE. In accordance with our observations, another group reported that drusen in AMD donor eyes sections contained increased levels of autophagic markers [63]. Moreover, dysregulated autophagy in RPE was recently associated with increased susceptibility to oxidative stress, and AMD [17, 18]. A recent study also reported increased flavoprotein fluorescence in nonexudative eyes with AMD, proposing mitochondrial dysfunction in AMD [64].

The RPE is constantly exposed to light-induced oxidative damage and oxidative stress has been proposed as an important factor in contributing to the development of AMD [4, 65]. Nonetheless, the mechanisms underlying the susceptibility to oxidative stress in AMD remains to be elucidated.

PGC-1α plays a major role in mitochondrial biogenesis and oxidative metabolism [66]. Its repression is associated with obesity, diabetes, neurodegeneration, and cardiomyopathy disorders [23–27]. A role for PGC-1α in determining light damage susceptibility and regulating normal and pathological angiogenesis in the retina has also been reported [24, 67]. A recent study proposed a role for PGC-1α in induction of human RPE oxidative metabolism and antioxidant capacity [29]. However, to date the role of PGC-1α in the pathophysiology of AMD is largely unknown.

To investigate the underlying mechanisms related to the observed phenotypes in our cells, we analyzed the *PGC-1α* expression. Our data showed lower *PGC*-*1α* expression in AMD iPSC-RPE cells compared to normal iPSC-RPE in accordance with the phenotypic disease state of the cells.

**Fig. 7:**
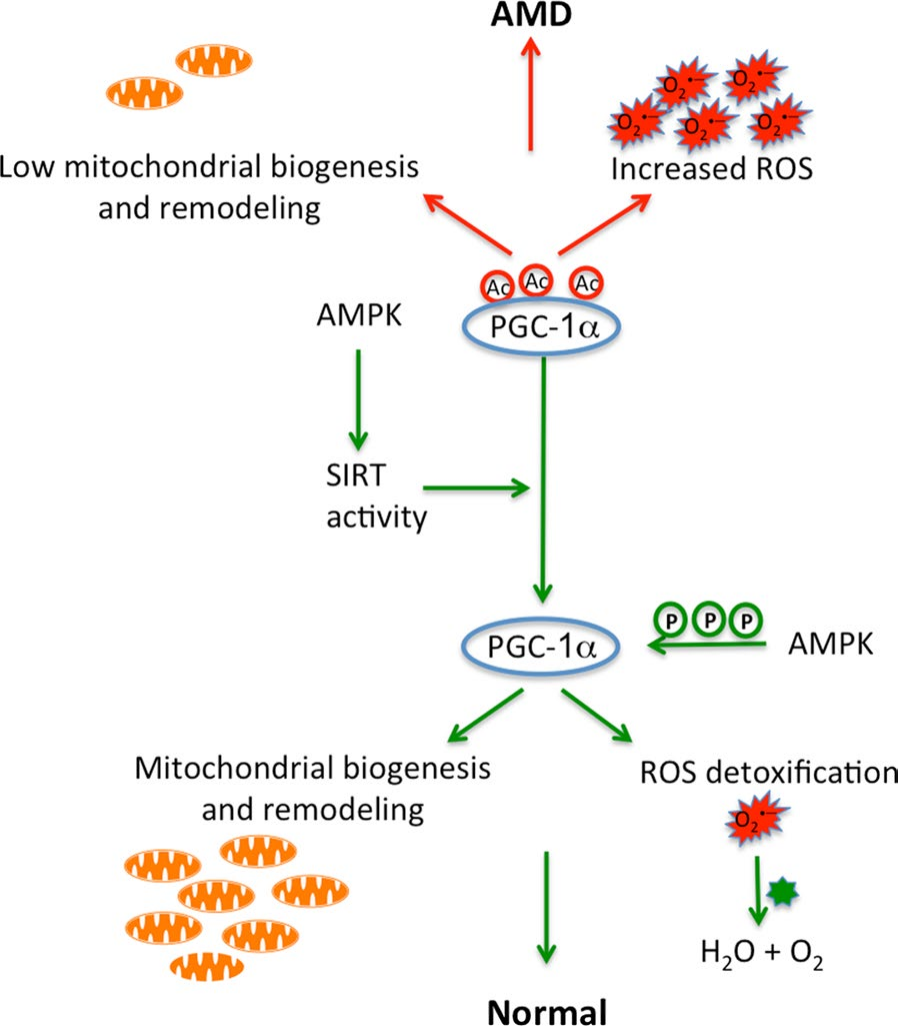
The role of SIRT1/PGC-1α repression in the pathophysiology of AMD. SIRT1 deacetylates and activates PGC-1α. AMPK increases SIRT1 activity and directly phosphorylates and activates PGC-1α. The reduction in SIRT1 activity would reduce deacetylation rate of PGC-1α. Hyperacetylation decreases PGC-1α activity which translates to lower mitochondrial content and activity, lowered mitochondrial respiratory capacity, lowered ROS detoxification and increased ROS production, contributing to the pathophysiology of AMD. *Ac* acetylation, *p* phosphorylation

We further analyzed SIRT1 expression in AMD iPSC-RPE and normal iPSC-RPE. Accordingly, SIRT1 protein levels were reduced in the AMD RPE-iPSC-RPE and AMD Skin-iPSC-RPE as compared to normal RPE-iPSC-RPE. SIRT1 is known to directly affect PGC-1α activity through phosphorylation and deacetylation [23]. It is also know that AMPK activation increases *PGC*-*1α* expression, and activates PGC-1α by direct phosphorylation [68]. AMPK activation also induces SIRT1-mediated PGC-1α deacetylation [69]. AMPK activation is triggered by increases in cellular AMP/ATP ratio [61]. Inactive AMPK results in lower autophagy dynamics causing accumulation of lipids and unwanted materials in the cells. Inactive AMPK also induces PGC-1α inactivation and results in lower mitochondrial biogenesis and turnover affecting mitochondrial activity. Increased total ATP caused by glycolysis that we observed in the AMD iPSC-RPE cells could result in AMPK inactivation and consequently lower the *PGC*-*1α* expression and activation. Based on our observations we hypothesize that repressed AMPK / SIRT1 / PGC-1α pathway could affect mitochondrial biogenesis and remodeling, causing increased ROS production, leading to RPE and retinal degeneration in AMD. Figure 7, summarizes our hypothesis.

Our data suggest involvement of SIRT1/PGC-1α pathway in the pathophysiology of AMD and open new avenues for development of novel and targeted drugs for treatment of AMD.

## Conclusions

Our study identified morphological and functional differences between normal iPSC-RPE and AMD iPSC-RPE generated from RPE of healthy donors and RPE of donors with AMD. We also observed that the iPSC-RPE generated from skin biopsy of a dry AMD patient exhibit the same disease cellular phenotypes and therefore can be used for in vitro disease modeling. The morphological changes observed in AMD RPE-iPSC-RPE and Skin-iPSC-RPE consist of disintegrated mitochondria, increased numbers of autophagosomes, and lipid droplets. We further performed functional analyses of AMD RPE-iPSC-RPE and AMD Skin-iPSC-RPE and observed increased susceptibility to oxidative stress, higher ROS production under oxidative stress, decreased mitochondrial activity, inability to increase *SOD2* expression under oxidative stress conditions, and higher cytoplasmic glycogen as compared to normal RPE-iPSC-RPE. In addition, we observed lower SIRT1 levels and lower *PGC*-*1α* expression in AMD iPSC-RPE as opposed to normal iPSC-RPE.

In summary, our study suggests that repressed SIRT1/PGC-1α causing impaired mitochondrial activity in RPE as responsible mechanisms for the disease cellular phenotypes that we observed in AMD RPE. Our data provide new insight in molecular mechanisms of AMD and could be used for development of potential therapeutic interventions.

## Additional file

**Additional file 1.** Additional figures and tables.

## Abbreviations

AMD: age-related macular degeneration
RPE: retinal pigment epithelium
iPSCs: induced pluripotent stem cells
EM: electron microscopy
ROS: reactive oxygen species
BSA: bovine albumin serum
TBST: (Tris buffered saline, Tween 20)
SOD2: superoxide dismutase 2
SNP: single nucleotide polymorphisms
POS: photoreceptor outer segment
mtDNA: mitochondrial DNA
NV: neovascular
ETC: electron transport chain
PGC-1α: peroxisome proliferator-activated receptor gamma coactivator 1-alpha
SIRT1: Sirtulin1 (silent information regulator T1)
AMPK: AMP-activated protein kinase

## References

[1] Gehrs KM, Anderson DH, Johnson LV, Hageman GS (2006). Age-related macular degeneration—emerging pathogenetic and therapeutic concepts. Ann Med.

[2] Bok D (1993). The retinal pigment epithelium: a versatile partner in vision. J Cell Sci Suppl.

[3] Boulton M, Dayhaw-Barker P (2001). The role of the retinal pigment epithelium: topographical variation and ageing changes. Eye (Lond).

[4] Bowes Rickman C, Farsiu S, Toth CA, Klingeborn M (2013). Dry age-related macular degeneration: mechanisms, therapeutic targets, and imaging. Invest Ophthalmol Vis Sci.

[5] Nowak JZ (2006). Age-related macular degeneration (AMD): pathogenesis and therapy. Pharmacol Rep.

[6] Abdelsalam A, Del Priore L, Zarbin MA (1999). Drusen in age-related macular degeneration: pathogenesis, natural course, and laser photocoagulation-induced regression. Surv Ophthalmol.

[7] Ferris FL, Fine SL, Hyman L (1984). Age-related macular degeneration and blindness due to neovascular maculopathy. Arch Ophthalmol.

[8] Lim LS, Mitchell P, Seddon JM, Holz FG, Wong TY (2012). Age-related macular degeneration. Lancet.

[9] Takahashi K, Tanabe K, Ohnuki M, Narita M, Ichisaka T, Tomoda K (2007). Induction of pluripotent stem cells from adult human fibroblasts by defined factors. Cell.

[10] Takahashi K, Yamanaka S (2006). Induction of pluripotent stem cells from mouse embryonic and adult fibroblast cultures by defined factors. Cell.

[11] Onder TT, Daley GQ (2012). New lessons learned from disease modeling with induced pluripotent stem cells. Curr Opin Genet Dev.

[12] Carr AJ, Vugler AA, Hikita ST, Lawrence JM, Gias C, Chen LL (2009). Protective effects of human iPS-derived retinal pigment epithelium cell transplantation in the retinal dystrophic rat. Plos ONE.

[13] Buchholz DE, Hikita ST, Rowland TJ, Friedrich AM, Hinman CR, Johnson LV (2009). Derivation of functional retinal pigmented epithelium from induced pluripotent stem cells. Stem Cells.

[14] Kokkinaki M, Sahibzada N, Golestaneh N (2011). Human iPS-derived retinal pigment epithelium (RPE) cells exhibit ion transport, membrane potential, polarized VEGF secretion and gene expression pattern similar to native RPE. Stem Cells.

[15] Yang J, Li Y, Chan L, Tsai YT, Wu WH, Nguyen HV (2014). Validation of genome-wide association study (GWAS)-identified disease risk alleles with patient-specific stem cell lines. Hum Mol Genet.

[16] Chang YC, Chang WC, Hung KH, Yang DM, Cheng YH, Liao YW (2014). The generation of induced pluripotent stem cells for macular degeneration as a drug screening platform: identification of curcumin as a protective agent for retinal pigment epithelial cells against oxidative stress. Front Aging Neurosci.

[17] Mitter SK, Song C, Qi X, Mao H, Rao H, Akin D (2014). Dysregulated autophagy in the RPE is associated with increased susceptibility to oxidative stress and AMD. Autophagy.

[18] Viiri J, Amadio M, Marchesi N, Hyttinen JM, Kivinen N, Sironen R (2013). Autophagy activation clears ELAVL1/HuR-mediated accumulation of SQSTM1/p62 during proteasomal inhibition in human retinal pigment epithelial cells. Plos ONE.

[19] Piippo N, Korkmaz A, Hytti M, Kinnunen K, Salminen A, Atalay M (2014). Decline in cellular clearance systems induces inflammasome signaling in human ARPE-19 cells. Biochim Biophys Acta.

[20] Terluk MR, Kapphahn RJ, Soukup LM, Gong H, Gallardo C, Montezuma SR (2015). Investigating mitochondria as a target for treating age-related macular degeneration. J Neurosci.

[21] Karunadharma PP, Nordgaard CL, Olsen TW, Ferrington DA (2010). Mitochondrial DNA damage as a potential mechanism for age-related macular degeneration. Invest Ophthalmol Vis Sci.

[22] Jarrett SG, Rohrer B, Perron NR, Beeson C, Boulton ME (2013). Assessment of mitochondrial damage in retinal cells and tissues using quantitative polymerase chain reaction for mitochondrial DNA damage and extracellular flux assay for mitochondrial respiration activity. Methods Mol Biol.

[23] Canto C, Auwerx J (2009). PGC-1 alpha, SIRT1 and AMPK, an energy sensing network that controls energy expenditure. Curr Opin Lipidol.

[24] Egger A, Samardzija M, Sothilingam V, Tanimoto N, Lange C, Salatino S (2012). PGC-1α determines light damage susceptibility of the murine retina. Plos ONE.

[25] Lelliott CJ, Medina-Gomez G, Petrovic N, Kis A, Feldmann HM, Bjursell M (2006). Ablation of PGC-1 beta results in defective mitochondrial activity, thermogenesis, hepatic function, and cardiac performance. PLoS Biol.

[26] Crunkhorn S, Dearie F, Mantzoros C, Gami H, da Silva WS, Espinoza D (2007). Peroxisome proliferator activator receptor gamma coactivator-1 expression is reduced in obesity: potential pathogenic role of saturated fatty acids and p38 mitogen-activated protein kinase activation. J Biol Chem.

[27] Jones AW, Yao Z, Vicencio JM, Karkucinska-Wieckowska A, Szabadkai G (2012). PGC-1 family coactivators and cell fate: roles in cancer, neurodegeneration, cardiovascular disease and retrograde mitochondria-nucleus signalling. Mitochondrion.

[28] SanGiovanni JP, Chen J, Sapieha P, Aderman CM, Stahl A, Clemons TE (2013). DNA sequence variants in PPARGC1A, a gene encoding a coactivator of the omega-3 LCPUFA sensing PPAR-RXR transcription complex, are associated with NV AMD and AMD-associated loci in genes of complement and VEGF signaling pathways. Plos ONE.

[29] Iacovelli J, Rowe GC, Khadka A, Diaz-Aguilar D, Spencer C, Arany Z (2016). PGC-1alpha induces human RPE oxidative metabolism and antioxidant capacity. Invest Ophthalmol Vis Sci.

[30] St-Pierre J, Lin J, Krauss S, Tarr PT, Yang R, Newgard CB (2003). Bioenergetic analysis of peroxisome proliferator-activated receptor gamma coactivators 1alpha and 1beta (PGC-1alpha and PGC-1beta) in muscle cells. J Biol Chem.

[31] St-Pierre J, Drori S, Uldry M, Silvaggi JM, Rhee J, Jager S (2006). Suppression of reactive oxygen species and neurodegeneration by the PGC-1 transcriptional coactivators. Cell.

[32] Vainshtein A, Desjardins EM, Armani A, Sandri M, Hood DA (2015). PGC-1alpha modulates denervation-induced mitophagy in skeletal muscle. Skelet Muscle.

[33] Vainshtein A, Tryon LD, Pauly M, Hood DA (2015). Role of PGC-1alpha during acute exercise-induced autophagy and mitophagy in skeletal muscle. Am J Physiol Cell Physiol.

[34] Valle I, Alvarez-Barrientos A, Arza E, Lamas S, Monsalve M (2005). PGC-1alpha regulates the mitochondrial antioxidant defense system in vascular endothelial cells. Cardiovasc Res.

[35] Shore D, Squire M, Nasmyth KA (1984). Characterization of two genes required for the position-effect control of yeast mating-type genes. EMBO J.

[36] Jeninga EH, Schoonjans K, Auwerx J (2010). Reversible acetylation of PGC-1: connecting energy sensors and effectors to guarantee metabolic flexibility. Oncogene.

[37] An E, Sen S, Park SK, Gordish-Dressman H, Hathout Y (2010). Identification of novel substrates for the serine protease HTRA1 in the human RPE secretome. Invest Ophthalmol Vis Sci.

[38] Sonoda S, Spee C, Barron E, Ryan SJ, Kannan R, Hinton DR (2009). A protocol for the culture and differentiation of highly polarized human retinal pigment epithelial cells. Nat Protoc.

[39] Villegas J, McPhaul M. Establishment and culture of human skin fibroblasts. In: Frederick M Ausubel, et al, editors. Current protocols in molecular biology. 2005. doi: 10.1002/0471142727.mb2803s71. 10.1002/0471142727.mb2803s71 18265368

[40] Poliakov E, Strunnikova NV, Jiang JK, Martinez B, Parikh T, Lakkaraju A (2014). Multiple A2E treatments lead to melanization of rod outer segment-challenged ARPE-19 cells. Mol Vis.

[41] Finnemann SC, Bonilha VL, Marmorstein AD, Rodriguez-Boulan E (1997). Phagocytosis of rod outer segments by retinal pigment epithelial cells requires alpha(v)beta5 integrin for binding but not for internalization. Proc Natl Acad of Sci USA.

[42] Schreck RR, Disteche C. Karyotyping. Curr Protoc Hum Genet. 2001. doi:10.1002/0471142905.hga04as18**(Appendix 4:Appendix 4A) **. 10.1002/0471142905.hga04as18 18428228

[43] Stone JF, Sandberg AA (1995). Sex chromosome aneuploidy and aging. Mutat Res.

[44] Na J, Baker D, Zhang J, Andrews PW, Barbaric I (2014). Aneuploidy in pluripotent stem cells and implications for cancerous transformation. Protein Cell.

[45] McCord JM, Edeas MA (2005). SOD, oxidative stress and human pathologies: a brief history and a future vision. Biomed Pharmacother.

[46] Murphy MP (2013). Mitochondrial dysfunction indirectly elevates ROS production by the endoplasmic reticulum. Cell Metab.

[47] Seo YH, Jung HJ, Shin HT, Kim YM, Yim H, Chung HY (2008). Enhanced glycogenesis is involved in cellular senescence via GSK3/GS modulation. Aging Cell.

[48] Lee SJ, Zhang J, Choi AM, Kim HP (2013). Mitochondrial dysfunction induces formation of lipid droplets as a generalized response to stress. Oxid Med Cell Longev.

[49] Boren J, Brindle KM (2012). Apoptosis-induced mitochondrial dysfunction causes cytoplasmic lipid droplet formation. Cell Death Differ.

[50] Bai P, Canto C, Brunyanszki A, Huber A, Szanto M, Cen Y (2011). PARP-2 regulates SIRT1 expression and whole-body energy expenditure. Cell Metab.

[51] Rein DB, Wittenborn JS, Zhang X, Honeycutt AA, Lesesne SB, Saaddine J (2009). Forecasting age-related macular degeneration through the year 2050: the potential impact of new treatments. Arch Ophthalmol.

[52] Cherry AB, Daley GQ (2012). Reprogramming cellular identity for regenerative medicine. Cell.

[53] Bar-Nur O, Russ HA, Efrat S, Benvenisty N (2011). Epigenetic memory and preferential lineage-specific differentiation in induced pluripotent stem cells derived from human pancreatic islet beta cells. Cell Stem Cell.

[54] Hu Q, Friedrich AM, Johnson LV, Clegg DO (2010). Memory in induced pluripotent stem cells: reprogrammed human retinal-pigmented epithelial cells show tendency for spontaneous redifferentiation. Stem Cells.

[55] Kim K, Doi A, Wen B, Ng K, Zhao R, Cahan P (2010). Epigenetic memory in induced pluripotent stem cells. Nature.

[56] Sullivan GJ, Bai Y, Fletcher J, Wilmut I (2010). Induced pluripotent stem cells: epigenetic memories and practical implications. Mol Hum Reprod.

[57] Polo JM, Liu S, Figueroa ME, Kulalert W, Eminli S, Tan KY (2010). Cell type of origin influences the molecular and functional properties of mouse induced pluripotent stem cells. Nat Biotechnol.

[58] Rose S, Frye RE, Slattery J, Wynne R, Tippett M, Pavliv O (2014). Oxidative stress induces mitochondrial dysfunction in a subset of autism lymphoblastoid cell lines in a well-matched case control cohort. Plos ONE.

[59] Sena LA, Chandel NS (2012). Physiological roles of mitochondrial reactive oxygen species. Mol Cell.

[60] Petersen KF, Befroy D, Dufour S, Dziura J, Ariyan C, Rothman DL (2003). Mitochondrial dysfunction in the elderly: possible role in insulin resistance. Science.

[61] Hardie DG, Ross FA, Hawley SA (2012). AMPK: a nutrient and energy sensor that maintains energy homeostasis. Nat Rev Mol Cell Biol.

[62] Kim J, Kundu M, Viollet B, Guan KL (2011). AMPK and mTOR regulate autophagy through direct phosphorylation of Ulk1. Nat Cell Biol.

[63] Wang AL, Lukas TJ, Yuan M, Du N, Tso MO, Neufeld AH (2009). Autophagy and exosomes in the aged retinal pigment epithelium: possible relevance to drusen formation and age-related macular degeneration. PLoS ONE.

[64] Field MG, Comer GM, Kawaji T, Petty HR, Elner VM (2012). Noninvasive imaging of mitochondrial dysfunction in dry age-related macular degeneration. Ophthalmic Surg Lasers Imaging.

[65] Beatty S, Koh H, Phil M, Henson D, Boulton M (2000). The role of oxidative stress in the pathogenesis of age-related macular degeneration. Surv Ophthalmol.

[66] Liang H, Ward WF (2006). PGC-1alpha: a key regulator of energy metabolism. Adv Physiol Educ.

[67] Saint-Geniez M, Jiang A, Abend S, Liu L, Sweigard H, Connor KM (2013). PGC-1alpha regulates normal and pathological angiogenesis in the retina. Am J Pathol.

[68] Jager S, Handschin C, St-Pierre J, Spiegelman BM (2007). AMP-activated protein kinase (AMPK) action in skeletal muscle via direct phosphorylation of PGC-1alpha. Proc Natl Acad Sci USA.

[69] Canto C, Gerhart-Hines Z, Feige JN, Lagouge M, Noriega L, Milne JC (2009). AMPK regulates energy expenditure by modulating NAD+ metabolism and SIRT1 activity. Nature.

