# Supplementary material for: Repressed SIRT1/PGC-1α pathway and mitochondrial disintegration in iPSC-derived RPE disease model of age-related macular degeneration
Source: J Transl Med. 2016 Dec 20;14:344. doi: 10.1186/s12967-016-1101-8 (PMC5175395; doi:10.1186/s12967-016-1101-8)
Supplement: Supplementary file 1 — Additional file 1. Additional figures and tables. [file 12967_2016_1101_MOESM1_ESM.doc]

**SUPPLEMENTARY FIGUREs**

Submitted separately

**SUPPLEMENTARY LEGENDS**

**Supplementary Figure S1.**

**
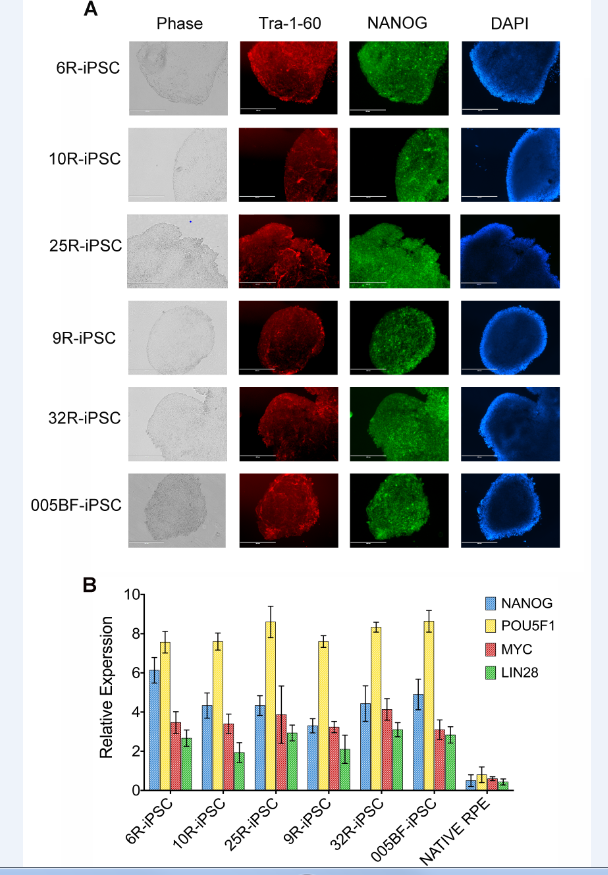
**

**Generation and characterization of RPE-iPSCs and Skin fibroblast iPSCs**

**(A**) Phase contrast image and immunostaining of iPSCs with antibodies for Tra-1-60 (AlexaFluor 594nm; red fluorescence), NANOG (AlexaFluor 488nm; green fluorescence), and DAPI (blue fluorescence). All scale bars represent 400um.

(**B**) Gene expression of iPSCs**.** Pluripotency of AMD and control iPSCs were confirmed using RT-PCR for *NANOG*, *POU5F1*, *cMYC* and *LiN28*. *GAPDH* was used to normalize each sample and the relative gene expression of gene is depicted by bar graph.

**Supplementary Figure S2.**

**
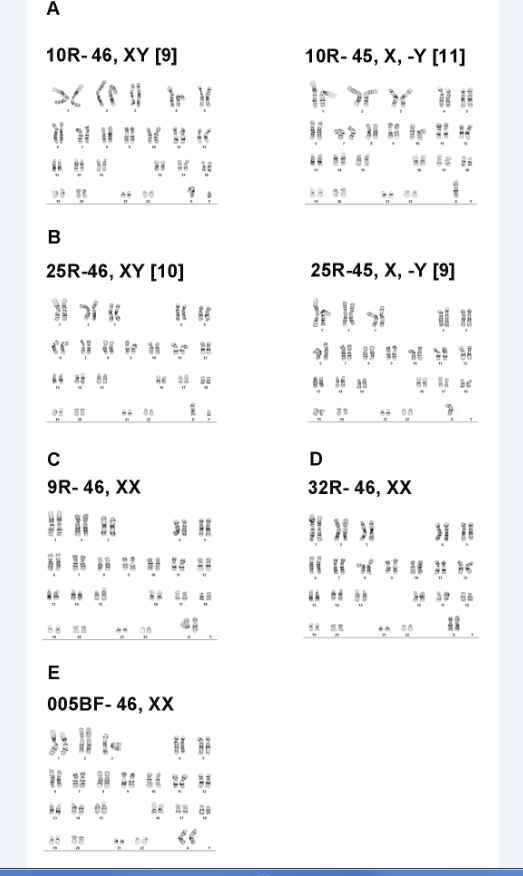
**

**G-band karyotyping of the RPE-iPSC-RPE and Skin-iPSC-RPE.**

(**A**): Karyotyping of RPE from a male healthy donor #10R showing normal somatic chromosomes and missing Y chromosome in approximately 50% of the tested cells.

(**B**): Karyotyping of RPE from a male healthy donor #25R showing normal somatic chromosomes and missing Y chromosome in approximately 50% of the tested cells.

(**C**) Karyotyping of RPE from a female AMD donor #9R showing normal chromosome integrity.

(**D**) Karyotyping of RPE from a female AMD donor #32R showing normal chromosome integrity.

(**E**) Karyotyping of skin fibroblasts from a female AMD patient #005BF showing normal chromosome integrity.

**Supplementary Figure S3.**

**
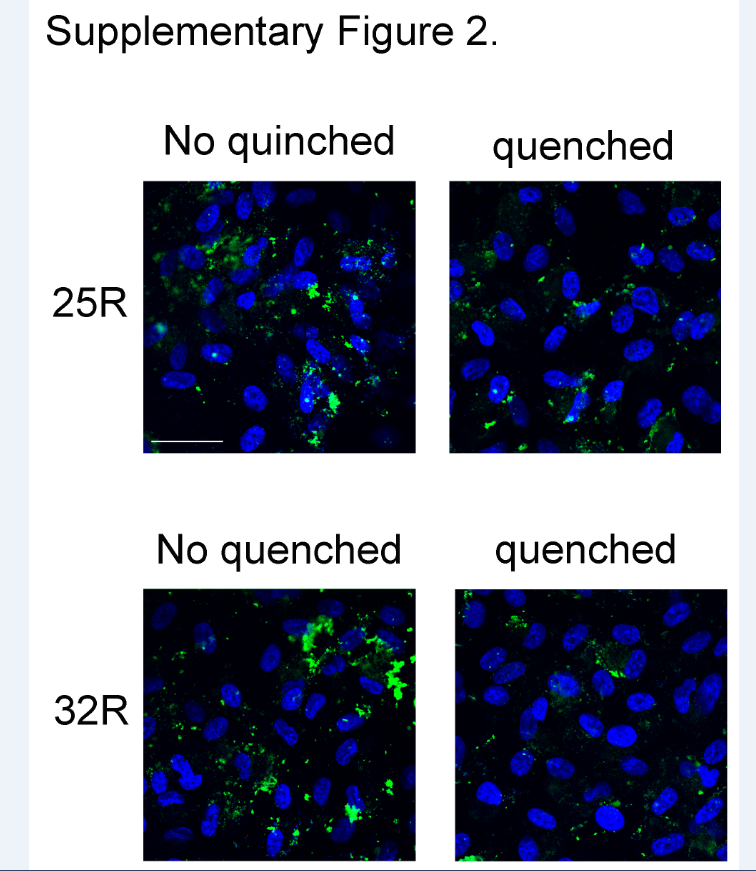
**

Representative images of phagocytosis in normal RPE (25R) and AMD RPE (32R). The left panels show the FITC conjugated-POS without Trypan blue quenching and the right panel shows the phagocytosis of POS after Trypan blue treatment, representing internalized POS.

**Supplementary Table S1.**

**Primers used in this study**:

| **Primer Name** | **Primer Sequence (5'-3')** |
| --- | --- |
| POU5F1 FOR  POU5F1 REV | CTGTCTCCGTCACCACTCTG  TGTGTTCCCAATTCCTTCCTTAG |
| NANOG FOR  NANOG REV | CCTGAACCTCAGCTACAAACAGGT  CACACCATTGCTATTCTTCGGCCA |
| LIN28 FOR  LIN28 REV | CGGGCATCTGTAAGTGGTTC  GTAGGTTGGCTTTCCCTGTG |
| MYC FOR  MYC REV | AGCGACTCTGAGGAGGAACAAGAA  CGTAGTTGTGCTGATGTGTGGAGA |
| RPE65 FOR  RPE65 REV | CTGTCCTCGCCGCTCACAGC  GCAGGAGGG CTTGCCCATCAA |
| CRALBP FOR  CRALBP REV | TGTGGGCGGTGAGAAGTTCCTTAT  TCTTCTCTTCCACTTGCAGGCTGT |
| SILV FOR  SILV REV | GCCTGGCAGTGGTCAGCACC  CGGGGTAGACGCAGCCAGTGA |
| PAX6 FOR  PAX6 REV | ACCAATTCCACAACCCACCACA  TGCCCATTGGCTGACTGTTCAT |
| MITF FOR  MITF REV | TTCACGAGCGTCCTGTATGCAGAT  AGTTTCCCGAGACAGGCAACGTAT |
| OTX2 FOR  OTX2 REV | AAGTTCCACTGCTCCAAACCCA  ACTCAGCCCATTGACTGCGTAA |
| RAX FOR  RAX REV | TTTCACCACGTACCAGCTGCAT  TGCAGCTTCATGGAGGACACTT |
| SIX3 FOR  SIX3 REV | TCACTCCCACACAAGTAGGCAA  CGGCCTTGGCTATCATACATCACA |
| VEGFA FOR  VEGFA REV | AAGGAGGAGGGCAGAATCAT  ATCTGCATGGTGATGTTGGA |
| LHX2 FOR  LHX2 REV | TGTTTCAGCAAGGACGGTAGCA  GCACGTGAAGCAGTTGAGGTGATA |
| PEDF FOR  PEDF REV | TGTGCAGGCTTAGAGGGA  GTTCACGGGGACTTTGAA |
| SOD2 | Validated PCR Primer Set # VHPS-8741 from RealTimePrimers.com |
| PGC1α FOR  PGC1α REV | AGAGGTAACAGCCTCCAGTGA  AACCCCATGCCATCCATCTT |
| GAPDH FOR  GAPDH REV | GAGTCAACGGATTTGGTCGTAT  AATGAAGGGGTCATTGATGG |

| **Supplementary Table S2**. Antibodies used in this study   |  | | |  | | --- | --- | --- | --- | | **Antibody Host Company Dilution** |  |  |  | |  | **Dilution** | |  |  | |  | | *Primary Antibodies used in immunostaining*  Tra-1-60-AF488 Mouse Life Technologies 1/100  Nanog Goat Novus Biologicals 1/50  ZO-1 Mouse Life Technologies 1/100  RPE-65 Rabbit Generous gift from 1/100  Dr. Redmond lab  Occludin Rabbit Invitrogen 1/500  Bestrophin Mouse Abcam 1/500  SIRT-1 Rabbit Cell Signaling 1/1000  **Antibody Host Company Dilution**  AlexaFluor594- Goat Invitrogen 1/1000  anti-mouse | | |  | | AlexaFluor488- Donkey Invitrogen 1/1000  anti-goat |  |  |  | |  |  |  |  | | AlexaFluor488- Donkey Invitrogen 1/1000  anti-rabbit |  |  |  | | *Secondary Antibodies, horseradish peroxidase (HRP)- conjugated, used in Western blot* |  |  |  | |  |  |  |  | | anti-rabbit-HRP Donkey Jackson Immunoresearch 1/10,000 |  | Jackson Immunoresearch | 1/10,000 |   **Other reagents**:  POS-FITC conjugation: Fluorescein-5-Isothiocyanate (FITC 'Isomer I')  (Molecular Probes, Life Technologies)    DAPI: NucBlue Fixed Cell Stain ReadyProbes reagent DAPI Special Formulation  (Molecular Probes, Life Technologies) |  |  | 1/100 |
| --- | --- | --- | --- | --- | --- | --- | --- | --- | --- | --- | --- | --- | --- | --- | --- | --- | --- | --- | --- | --- | --- | --- | --- | --- | --- | --- | --- | --- | --- | --- | --- | --- | --- | --- | --- | --- | --- | --- | --- | --- | --- | --- | --- | --- |
|  |  |  |  |
